# An evaluation survey of traditional Chinese medicine learning among international students majoring in conventional medicine: a study from a university in China

**DOI:** 10.1186/s12906-020-03174-1

**Published:** 2021-01-07

**Authors:** Fan Qu, Qing Zhang, Minchen Dai, Yijing He, Jiaqi Wu, Xian Zhang, Yuhang Zhu, Ying’er Gu, Fangfang Wang, Xiangrong Xu

**Affiliations:** 1 Women’s Hospital, School of Medicine, Zhejiang University, 1 Xueshi Road, Hangzhou, 310006 Zhejiang China; 2 The Second Clinical Medical College, Zhejiang Chinese Medical University, Hangzhou, 310053 China

**Keywords:** Traditional Chinese medicine, Education, International undergraduates, Cross-sectional survey

## Abstract

**Background:**

Traditional Chinese medicine (TCM) has gained increasing acceptance and popularity by the global community. The current study aimed to investigate self-reported evaluations of learning TCM and opinions about TCM courses among undergraduate international students majoring in conventional medicine at a university in China.

**Methods:**

A cross-sectional survey was conducted at the Zhejiang University School of Medicine. A self-reported questionnaire was administered to international undergraduates who were enrolled in the TCM course during the 2018 and 2019 academic years (*n* = 157). The course employed a student-centered, multiform learning model. Demographic data and self-reported evaluations of TCM learning background and TCM learning course were obtained to conduct the analysis.

**Results:**

A total of 133 students responded to the questionnaire. Among the respondents, 21.0% had some TCM-related knowledge, and 51.1% were interested in learning TCM before the course. Ninety-six students (85.7%) were from Asia. Students from Thailand showed significantly more interest in learning TCM than did students from other Asian countries (*p* = 0.025). After the course, 77.2% of students agreed that the course had brought about many benefits, 86.4% were satisfied with the course content, and 77.3% were satisfied with the teaching method. Students expressed their willingness to further learn acupuncture and to obtain more skilled practice through more visualized learning methods.

**Conclusions:**

The majority of the international students we surveyed agreed that the TCM course improved their interest in and understanding of TCM. It is thus suggested that TCM education should be directed toward students’ learning barriers and needs.

**Supplementary Information:**

The online version contains supplementary material available at 10.1186/s12906-020-03174-1.

## Background

Traditional Chinese medicine (TCM) education is an important component of biomedical education in China [1]. The Chinese Ministry of Health stipulates that undergraduate medical universities must provide nationally standardized courses in TCM [2, 3]. In China, undergraduate curricula for TCM education cover fundamental courses in Chinese medicine, including the classics of TCM, fundamental courses of acupuncture and moxibustion, clinical practice, etc. [4]. Unlike modern biomedical healthcare, TCM has a unique philosophical foundation and a holistic system of theories and practices [5], which make the TCM curriculum important but challenging. In recent years, TCM has been making its way to many countries worldwide, and related higher education programs have been developed in various modes in different countries, which reflect national policies, acceptance and popularity toward TCM [4–8].

With many international students coming to China to learn medicine, Chinese medical institutions have attached importance to the internationalization of their education system. There are 52 medical schools that offer an English Bachelor of Medicine and Surgery degree, which is a medical education program specifically designed for international students who wish to pursue a medical bachelor’s degree in China [9, 10]. The internationalization trend of medical education undoubtedly affects the global nature of medical curricula and, more importantly, promotes idea exchanges between different cultures [9]. However, TCM education for international students is a challenging task for universities and teachers, especially for those responsible for TCM curricula in Western medical universities or schools. Differences in cultural backgrounds and teaching approaches, gaps in language, insufficient professionals and teaching materials, and the lack of communication tools and opportunities are the main barriers to international students understanding the abstract and obscure theories of TCM [11]. Moreover, the lack of a sufficient learning atmosphere and enthusiasm for TCM, preconceived notions of conventional medicine, limited didactic hours and insufficient skills practice are general problems related to TCM education in medical universities or schools [12].

Zhejiang University is one of China’s higher education institutions. It currently ranks among the top 10 universities on the Chinese mainland [13] and within the top 100 universities in the QS World University Rankings 2020 [14]. A total of more than 6300 students, including a cohort of 600 international students, are currently studying with its faculty of medicine, i.e., the Zhejiang University School of Medicine (ZUSM) [15]. The course titled “Basic Traditional Chinese Medicine” has been set up in ZUSM as one of the curriculum requirements of the Medical Bachelor, Bachelor of Surgery (MBBS) program [10]. All undergraduate international students in ZUSM major in conventional medicine, and the TCM course is a component of their program system. Since the 2018 academic year, the course has employed student-centered, multiform teaching methods. In this study, a questionnaire was used to investigate self-reported evaluations of learning TCM and opinions about TCM courses among undergraduate international students in ZUSM.

## Methods

### Study design and setting

The cross-sectional survey was conducted at Zhejiang University, Hangzhou, China. In the 2018–2019 academic year, the course titled “Basic Traditional Chinese Medicine” was reformed from the traditional teacher-centered lecture-based learning (LBL) method to a student-centered, multiform learning model. The general objective of this course is to introduce the fundamental theories and skills practice of TCM to undergraduate international students.

### Participants

Two consecutive cohorts of students attending the “Basic Traditional Chinese Medicine” course in 2018 and 2019 (*n* = 80 and *n* = 77, respectively, for a total of 157) were enrolled in this study. Informed consent and a voluntary questionnaire were administered to the participants during the last class.

### Course design and teaching methods

The “Basic Traditional Chinese Medicine” course is provided in the second academic year (in the preclinical phase) of the six-year MBBS program. It covers 32 lessons, for a total of 1440 mins completed in eight teaching weeks, including 24 theoretical lessons and 8 practical lessons. The theoretical lessons consist of six parts: an introduction to TCM and traditional Chinese philosophy; TCM etiology and pathomechanism; TCM diagnostics; TCM therapeutic principles and methods; TCM life nurturing; and modern research on TCM. The practical lessons are interspersed and coordinated with theoretical lessons and are designed to help the students practice the skills of acupuncture, moxibustion, massage, cupping, the identification of Chinese herbs, the four diagnostic methods of TCM, and TCM methods of life nurturing such as Taiji. Beyond the 32 lessons, the course also includes clinical practice at the Department of Chinese Integrative Medicine, Women’s Hospital, School of Medicine, Zhejiang University, China. The course employs various teaching methods, including LBL, flipped classroom, problem-based learning (PBL), team-based learning (TBL), case-based learning (CBL), skill practice, experiential teaching and visualized teaching. One noticeable feature is the application of the flipped classroom. In brief, teachers determine the learning arrangements and lecture about theoretical knowledge, while students are divided into groups and encouraged to watch videos or consult literature beyond the class boundaries to consolidate or self-learn TCM-related knowledge. The classroom then becomes a place where presentations and discussions are performed by students for half the class time. Interaction is increased by the use of the question-and-answer mode between teachers and students. Students are graded in light of their attendance, class assignments, class presentation and final essays.

### Data collection

An anonymous, self-administered questionnaire (shown as Additional file 1) was handed out during the last theoretical lesson, which was also the last lesson of the course. Since there is no recognized authoritative questionnaire on TCM learning assessment, we designed the questionnaire according to the objective of the study. Prior to distribution, the questionnaire was sent to several TCM educators and students to determine its validity and consistency. The questionnaire comprised 21 questions, including 3 demographic data questions about the student’s age, gender and country of origin, and 18 close-ended questions consisting of 9 single choice and 9 multiple choice. The 9 single-choice questions aimed to investigate students’ self-reported evaluation of their knowledge, interest, understanding, benefits and satisfaction with TCM learning. Each question used a 4-point Likert scale, where 1 was ‘strongly disagree’, 2 was ‘disagree’, 3 was ‘agree’ and 4 was ‘strongly agree’. Then, we obtained a score for each question. The 9 multiple-choice questions aimed to investigate detailed information on the students’ opinions about the TCM courses, such as the course content and teaching methods. The students who agreed to participate in the study completed and returned the questionnaire. The average time needed to complete the questionnaire was 5 mins.

### Statistical analysis

The results of the questionnaire were entered and translated into digital format using Microsoft Office Excel Version 2011 (Microsoft Corporation, Redmond, Washington). Statistical analysis was performed using IBM SPSS (IBM Corp. Released 2012. IBM SPSS Statistics for Windows, Version 21.0. Armonk, NY: IBM Corp.). Descriptive information for variables was provided as the mean ± standard deviation (SD) or n (%), according to the nature of the variable. Variables were compared using one-way analysis of variance (ANOVA) at a statistical significance level of 0.05.

## Results

### Response rate and demographic characteristics

**Fig. 1:**
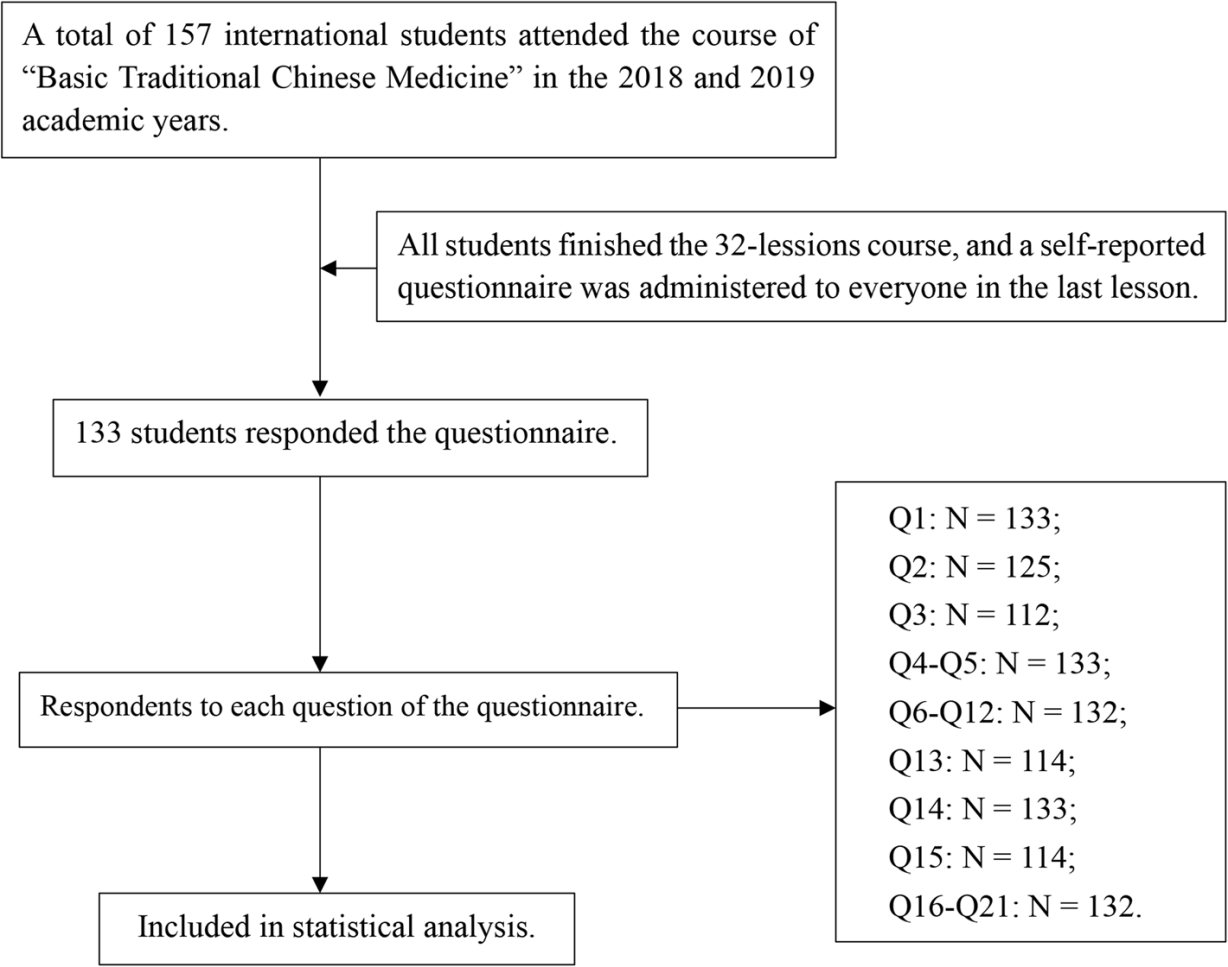
Flowchart of data collection and analysis

A total of 133 students responded to the questionnaire out of the 157 students who were enrolled in the “Basic Traditional Chinese Medicine” course during the 2018 and 2019 academic years. The response rate was 84.7%. The number of respondents for each question of the questionnaire is shown in Fig. 1. Among the participants, 52 (39.1%) were males and 81 (60.9%) were females. The response rate was higher among female students (92 students, 88.0%) than male students (65 students, 80.0%). In regard to the country of origin, 112 out of 133 students responded. Among the respondents, 96 students (85.7%) were from Asian countries, and the top five countries with large student populations were India 33 (29.5%), Thailand 27 (24.1%), Iraq 5 (4.5%), Bahrain 5 (4.5%) and Indonesia 5 (4.5%). The distribution of students by country of origin is shown in Table 1. The average age of the students (125 out of 133 students responded) was 21.90 ± 2.00 years old.

**Table 1:** Distribution of international students by country of origin (*n* = 112)

Country	Number	Percentage
India	33	29.5%
Thailand	27	24.1%
Iraq	5	4.5%
Bahrain	5	4.5%
Indonesia	5	4.5%
Syria	4	3.6%
Malaysia	3	2.7%
England	3	2.7%
Sudan	2	1.8%
Yemen	2	1.8%
Singapore	2	1.8%
Australia	2	1.8%
Iran	2	1.8%
Others^a^	17	15.2%

^a^Only 1 student for each country, i.e., Afghanistan, Bangladesh, Egypt, Germany

Ghana, Jamaica, Kazakhstan, Kenya, Kuwait, Pakistan, Russia, Rwanda, South Africa, Sri Lanka, United Arab Emirates, United States, and Zimbabwe

### Students’ self-reported evaluation of learning TCM

As shown in Table 2, among the respondents, 21.0% had some prior TCM-related knowledge, and 51.1% were interested in learning TCM before the course. Most students (84.1% of respondents) were able to follow the course content easily during the course. After the course finished, the percentage of students who agreed to have an improved perception, interest and understanding of TCM was 76.5, 66.7 and 61.4%, respectively. Moreover, 77.2% of students agreed that the course had brought about many benefits (score: 3.05 ± 0.57), 86.4% were satisfied with the course content (score: 3.30 ± 0.49), and 77.3% were satisfied with the teaching method (score: 3.20 ± 0.71) of the course.

**Table 2:** Students’ self-reported evaluations about learning TCM

Items	Proportion, N (%)				Score^**a**^, mean ± SD
	Strongly disagree	Disagree	Agree	Strongly agree	
**Evaluation of learning background**					
Before the course, you had some TCM-related knowledge. (*N* = 133)	26 (19.5)	79 (59.4)	24 (18.0)	4 (3.0)	2.05 ± 0.71
Before the course, you were interested in learning TCM. (N = 133)	18 (13.5)	47 (35.3)	47 (35.3)	21 (15.8)	2.53 ± 0.92
**Evaluation of the TCM learning course**					
You have understood well the content of the course. (*N* = 132)	2 (1.5)	19 (14.4)	84 (63.6)	27 (20.5)	3.03 ± 0.64
You have altered your perception of TCM. (N = 132)	4 (3.0)	27 (20.5)	75 (56.8)	26 (19.7)	2.93 ± 0.72
The course has improved your interest in TCM. (N = 132)	3 (2.3)	41 (31.1)	52 (39.4)	36 (27.3)	2.92 ± 0.82
You have a deeper understanding of TCM than before. (N = 132)	6 (4.5)	45 (34.1)	69 (52.3)	12 (9.1)	2.66 ± 0.71
The course brought you many benefits. (N = 132)	2 (1.5)	28 (21.2)	63 (47.7)	39 (29.5)	3.05 ± 0.76
You are satisfied with the content of the course. (N = 132)	0 (0.0)	18 (13.6)	57 (43.2)	57 (43.2)	3.30 ± 0.70
You are satisfied with the teaching methods of the course. (N = 132)	3 (2.3)	27 (20.5)	42 (31.8)	60 (45.5)	3.20 ± 0.85

^a^The score was obtained by a 4-point Likert scale, where 1 = strongly disagree, 2 = disagree, 3 = agree, 4 = strongly agree. *TCM* traditional Chinese medicine

As most students were from Asian countries, with India and Thailand being in the majority, the mean score of the self-reported evaluations of students from India, Thailand, other Asian countries and non-Asian countries were compared (shown in Table 3). The students from Thailand were significantly more interested in studying TCM than the students from other Asian countries, including India (*p* = 0.025).

**Table 3:** Self-reported evaluations of students from different regions about learning TCM

Items	Thailand (***N*** = 27)	India (***N*** = 33)	Other Asian countries (***N*** = 36)	Non-Asian countries (***N*** = 16)	***F***	***p***
**Evaluation of learning background**						
Before the course, you had some TCM-related knowledge.	2.11 ± 0.70	1.94 ± 0.70	2.06 ± 0.80	2.00 ± 0.52	0.321	0.810
Before the course, you were interested in learning TCM.	3.00 ± 0.79	2.48 ± 0.80^*^	2.33 ± 0.96^**^	2.56 ± 0.89	3.255	0.025
**Evaluation of the TCM learning course**						
You have understood well the content of the course.	3.00 ± 0.73	3.03 ± 0.73	3.06 ± 0.53	2.94 ± 0.68	0.127	0.944
You have altered your perception of TCM.	3.00 ± 0.68	2.94 ± 0.76	2.92 ± 0.81	2.75 ± 0.78	0.373	0.772
The course has improved your interest in TCM.	3.15 ± 0.82	2.88 ± 0.83	2.89 ± 0.71	2.81 ± 0.91	0.858	0.465
You have a deeper understanding of TCM than before.	2.63 ± 0.79	2.59 ± 0.71	2.64 ± 0.68	2.81 ± 0.66	0.351	0.788
The course brought you many benefits.	3.33 ± 0.83	3.15 ± 0.76	2.97 ± 0.61	2.81 ± 0.91	2.017	0.116
You are satisfied with the content of the course.	3.48 ± 0.64	3.36 ± 0.65	3.28 ± 0.70	3.19 ± 0.75	0.770	0.513
You are satisfied with the teaching methods of the course.	3.33 ± 0.78	3.33 ± 0.74	3.19 ± 0.92	2.94 ± 1.00	0.949	0.420

Data are presented as the mean ± SD. ^*^P < 0.05, ^**^P < 0.01 compared with students from Thailand by one-way ANOVA. *TCM* traditional Chinese medicine

### Reliability analysis of the questionnaire

We assessed the validity of 7 single-choice questions from the questionnaire that focused on the students’ self-reported evaluations of the TCM course. As shown in Table 4, the reliability coefficient (the value of Cronbach’s *α*) was 0.826, indicating that this subscale of the questionnaire (7 items) had good internal consistency. The Cronbach’s α values measured in 2018 and 2019 were both higher than 0.8, indicating that the reliability was stable across time.

**Table 4:** Reliability analysis of the subscale of the questionnaire

Year	N of observed values	N of items	Cronbach ***α***
2018	68	7	0.820
2019	63	7	0.838
Total	131	7	0.826

### Detailed information on students’ opinions about the TCM course

**Fig. 2:**
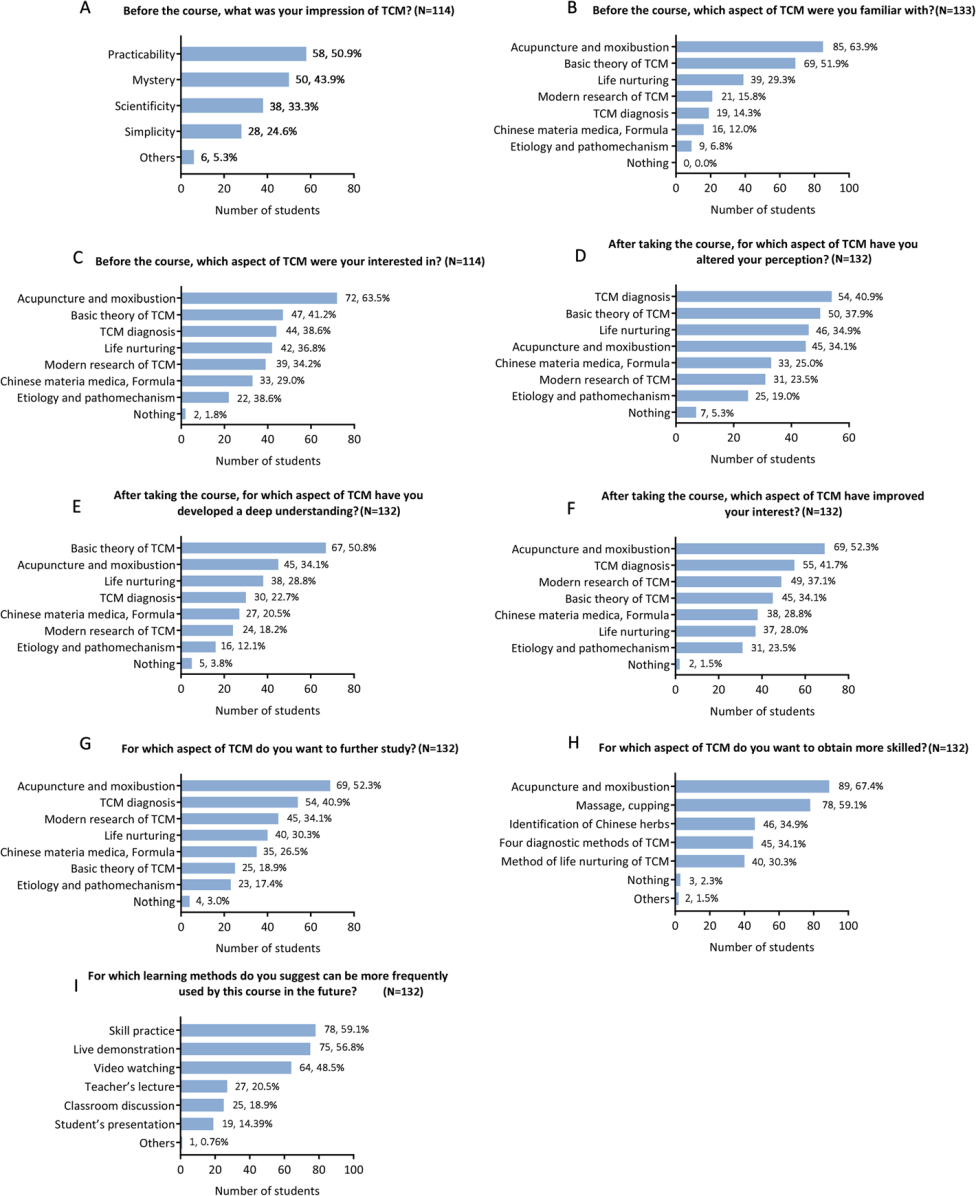
Detailed information of students’ opinions about the TCM course. TCM, traditional Chinese medicine

Before the course, 50.9 and 43.9% of the students’ impressions of TCM were focused on its practicability and mysterious nature, respectively (Fig. 2a); 63.9 and 51.9% of students had more knowledge about acupuncture and moxibustion and the basic theory of TCM, respectively (Fig. 2b); and 63.2% were most interested in acupuncture and moxibustion (Fig. 2c). After the course finished, 40.9, 37.9, and 34.9% of the students had altered their perception of TCM diagnosis, the basic theory of TCM, and life nurturing, respectively (Fig. 2d). In particular, 50.8% had a deeper understanding of the basic theory of TCM (Fig. 2e). Moreover, 52.3% of the students had improved their level of interest in regard to learning acupuncture and moxibustion (Fig. 2f); 52.3 and 40.9% of students wanted to continue their study of acupuncture and moxibustion and TCM diagnoses, respectively (Fig. 2g). In addition, 67.4 and 59.1% of the students wanted to engage in more skill practices primarily for acupuncture and moxibustion and for massage and cupping, respectively (Fig. 2h). Regarding the preferred teaching methods, 59.1, 56.8, and 48.5% of the students suggested the implementation of more skilled practice, live demonstrations and videos, respectively (Fig. 2i).

## Discussion

In this study, even though the undergraduate international students we surveyed were majoring in conventional medicine, a few students had basic prior knowledge of TCM, and half of them showed interest in TCM. After taking the TCM course, the majority of students agreed that the course had improved their interest, perception and understanding toward TCM, and they were satisfied with the course content and the teaching method. Students also expressed their willingness to further study acupuncture and obtain more skill practices in regard to acupuncture. Suggestions from students regarding the teaching methods included more skilled practice and more visualized learning methods.

TCM has gained increasing acceptance and popularity within the global community because of its alignment with biomedical models of professionalization, education, research and practice [6, 16]. Among the conventional health professions, acupuncture is a popular modality of complementary medicine, for example for chronic pain [17], although it could vary among settings, like clinical psychologists who would prefer to learn other complementary medicines [18]. Moreover, acupuncture is the only therapeutic method of TCM that the World Health Organization has identified for its effectiveness for numerous diseases [19], and it is the main field for modern research on TCM in countries other than China [20]. In such an international environment, it is no wonder that international students have at least a basic prior knowledge of TCM, mainly pertaining to acupuncture. The survey also showed that international students’ interest in learning TCM was mainly in relation to acupuncture and that they desired further study in regard to both fundamental theory and the skilled practice of acupuncture. Thus, in addition to satisfying the interest of international students in regard to learning acupuncture, TCM education should also emphasize the basic TCM theories, diagnostics and herbal remedies, as TCM comprises a whole system of medicine. Additionally, to alter the impression of foreign students that TCM is a “mystery”, the issue of how we can infuse evidence-based medicine into current TCM education and practice should be considered [21].

The respondents of this questionnaire-based study were mainly from Asia, among which more were from India and Thailand than from other countries. As students come from different ethnic groups and nations with a variety of cultural backgrounds and modes of thought [11], they have different perspectives and attitudes toward learning TCM. The students from Thailand showed more interest in learning TCM than did those from other Asian countries, which may be induced by the rapid spread and development of TCM in Thailand. In 2000, Thailand became the first country outside of China to legalize the practice of TCM [22]. Since 2003, higher education institutions in Thailand have developed partnerships with many TCM medical universities in China [23]. In 2016, the Confucius Institute of TCM was established in Thailand and has made great contributions to the spread of TCM [24]. These events in Thailand indicate that international collaboration and exchanges have strong potential in regard to promoting the internationalization of TCM education.

TCM education has been made a mandatory part of the medical education in most medical universities in China. Although challenges are perceived as persisting with regard to TCM education for international students in medical universities or schools in China [12, 25, 26], efforts to explore appropriate learning models have been made. A transformation from traditional didactics to those with a greater emphasis on PBL and CBL may be a welcomed reform in TCM education [27, 28]. The implementation of the flipped classroom, which integrates the application of online mini-video courses, teamwork, discussion, classroom presentation and a question-and-answer mode, has been put forward by scholars in the TCM education field [29]. In this study, an initial exploration was undertaken to reform the TCM course, which was mainly reflected in the transformation from the conventional teacher-centered LBL method to a student-centered multiform learning model. One previous study conducted in the medical school of Xi’an Jiaotong University in China surveyed international students’ barriers to and interests in learning TCM and collected their suggestions on the TCM curriculum; this study found that TCM education reformation was required and feasible [30]. Another study investigated the teaching effectiveness of multiple teaching methods applied in TCM education at Capital Medical University, a Western medical university in China, and found that the application of PBL, CBL and comparative teaching methods could improve students’ enthusiasm for learning TCM [28]. The present study was in accordance with the studies from other universities that have found that improving the teaching models is an important measure of reformation in regard to promoting TCM education [28, 30].

When international students were asked about their impression of TCM before the course, 50.9% agreed that it seemed practicable, and 43.9% considered it mysterious. A few students (21.0% of the respondents) reported that they had some prior TCM knowledge, and half of them (51.1% of the respondents) showed an interest in learning TCM. We investigated whether the course had positive impacts, and the majority of the students self-reported an improved perception, interest and understanding of TCM and that they had obtained benefits from the course. They also expressed satisfaction with the course. However, some students disagreed that the course had improved their interest in and understanding of TCM (33.4 and 38.6% of respondents, respectively). The reason for this outcome may be because the course is mainly theoretical and the theory of TCM itself is challenging. Moreover, similar to previous reports [1, 31], the international students we surveyed desired more skilled practice and live demonstrations, and acupuncture was the most preferred aspect to be learned and practiced by them. Thus, from the perspective that the attention of TCM education reform should be directed toward students’ learning barriers and needs, a better integration of theory and practice could be arranged in the TCM course design. It is also important to strengthen communication and interaction with international students, as well as to make the obscure TCM theories lively and accessible by using multimedia materials [25, 32].

This study has several limitations. First, although students from two academic years participated in the study, the sample size was small. The study was conducted at a single university, and the majority of the international students surveyed in this study were from Asian countries; thus, the responses did not reflect all international medical students in China. Second, the validity of the questionnaire has not been fully determined, and the self-administered questionnaire might lead to potential information bias from the participants. Third, we could not recruit a control group for the study due to the feasibility, and we also failed to have a pre- and post- evaluation to determine the effects of the educational intervention. Accordingly, in the future, we would consider a pretest/posttest study design on a continuous basis and across a larger sample of students to investigate the effects of the TCM course.

## Conclusions

In conclusion, international undergraduates majoring in conventional medicine in China seem to have a basic prior knowledge of and interest in TCM, especially in regard to acupuncture. The majority of the international students we surveyed agreed that the TCM course improved their interest in and understanding of TCM. They also showed satisfaction with the TCM course, which employed a student-centered, multiform learning model. It is suggested that TCM education reform should be directed toward students’ learning barriers and needs.

## Supplementary Information

**Additional file 1.** Title: Questionnaire for the “Basic Traditional Chinese Medicine” course. Description: Not applicable.

## Data Availability

The dataset supporting the conclusions of this article is available in the Mendeley Data repository, 10.17632/7rdppkyzy2.1

## Abbreviations

TCM: Traditional Chinese medicine
MBBS: Medical Bachelor, Bachelor of Surgery
ZUSM: Zhejiang University School of Medicine
LBL: Lecture-based learning
PBL: Problem-based learning
TBL: Team-based learning
CBL: Case-based learning
