# Supplementary figures and images for: An evaluation survey of traditional Chinese medicine learning among international students majoring in conventional medicine: a study from a university in China
Source: BMC Complement Med Ther. 2021 Jan 7;21:16. doi: 10.1186/s12906-020-03174-1 (PMC7791796; doi:10.1186/s12906-020-03174-1)

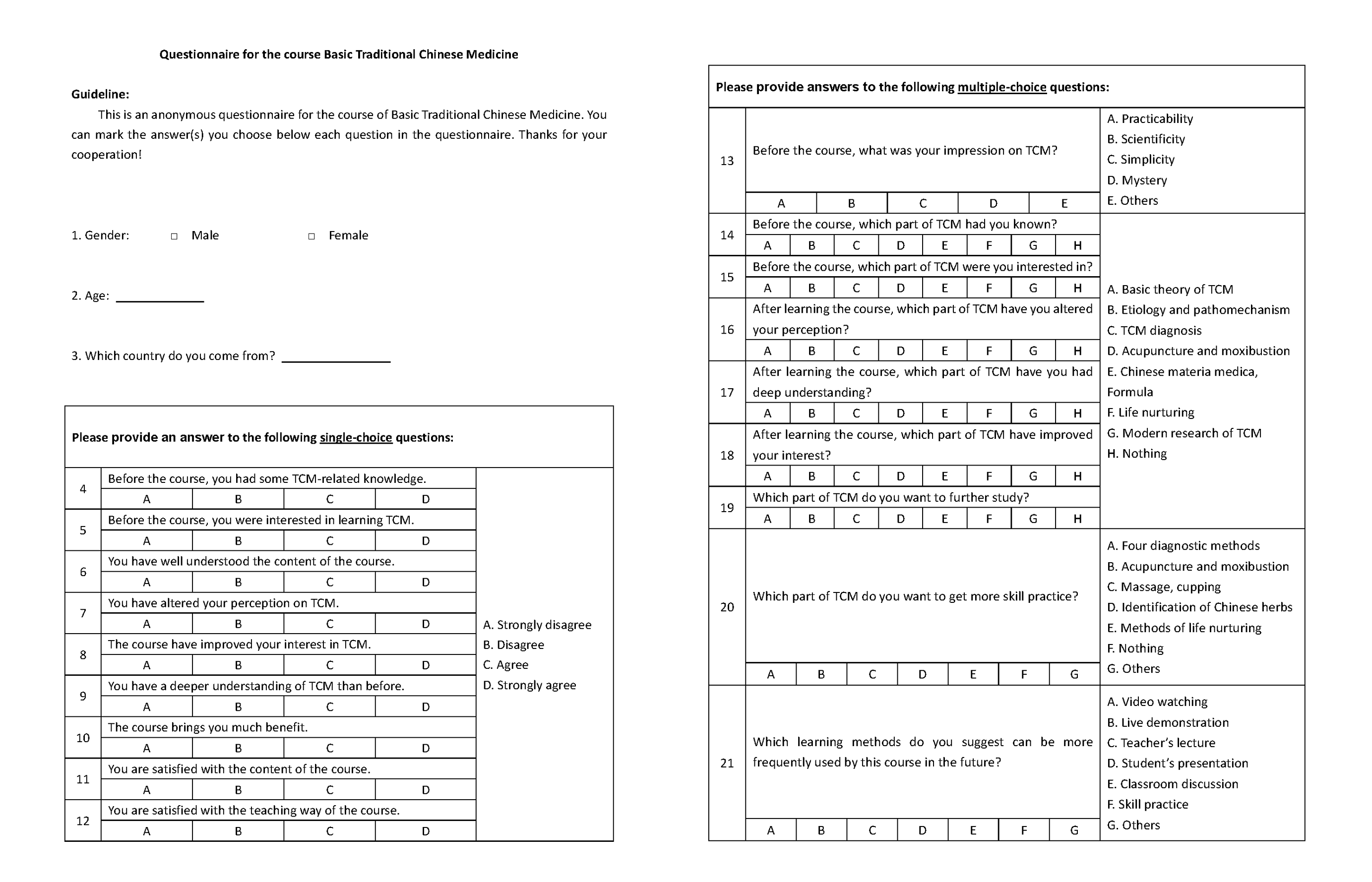

Supplement: Supplementary file 1 — Additional file 1. Title: Questionnaire for the “Basic Traditional Chinese Medicine” course. Description: Not applicable. [file 12906_2020_3174_MOESM1_ESM.tif]
